# Poly- and autoreactivity of HIV-1 bNAbs: implications for vaccine design

**DOI:** 10.1186/s12977-018-0435-0

**Published:** 2018-07-28

**Authors:** Joel Finney, Garnett Kelsoe

**Affiliations:** 1 Department of Immunology, Duke University, DUMC 3010, Durham, NC 27710 USA; 2 Human Vaccine Institute, Duke University, Durham, NC 27710 USA

**Keywords:** HIV-1, Antibody, Immunological tolerance, Polyreactivity, Autoreactivity

## Abstract

A central puzzle in HIV-1 research is the inability of vaccination or even infection to reliably elicit humoral responses against broadly neutralizing epitopes in the HIV-1 envelope protein. In infected individuals, broadly neutralizing antibodies (bNAbs) *do* arise in a substantial minority, but only after 2 or more years of chronic infection. All known bNAbs possess at least one of three traits: a high frequency of somatic hypermutation, a long third complementarity determining region in the antibody heavy chain (HCDR3), or significant poly- or autoreactivity. Collectively, these observations suggest a plausible explanation for the rarity of many types of bNAbs: namely, that their generation is blocked by immunological tolerance or immune response checkpoints, thereby mandating that B cells take a tortuous path of somatic evolution over several years to achieve broadly neutralizing activity. In this brief review, we discuss the evidence for this tolerance hypothesis, its implications for HIV-1 vaccine design, and potential ways to access normally forbidden compartments of the antibody repertoire by modulating or circumventing tolerance controls.

## Background

A principal aim of HIV-1 vaccine research is to elicit routinely broadly neutralizing antibodies (bNAbs), which target conserved, functionally important determinants on the HIV-1 envelope (Env) and consequently neutralize across viral clades [1]. However, bNAbs are difficult to elicit, arising in no more than 50% of HIV-1 patients, and only after 2 or more years of chronic infection [1–4]. Moreover, while vaccination with Env-derived antigens can initiate some bNAb lineages, substantive maturation of neutralization breadth and potency toward native viral isolates has not yet been achieved [5–9]. Several non-mutually exclusive hypotheses have been proposed to explain why generation of HIV-1 bNAbs is so challenging [10–12]. In this short review, we focus on the tolerance hypothesis [13], which posits that due to viral molecular mimicry of host structures, the B cells most fit to respond to broadly conserved, neutralizing epitopes are poly- or autoreactive, and have been removed from the repertoire by immunological tolerance controls [13–15].

**Fig. 1:**
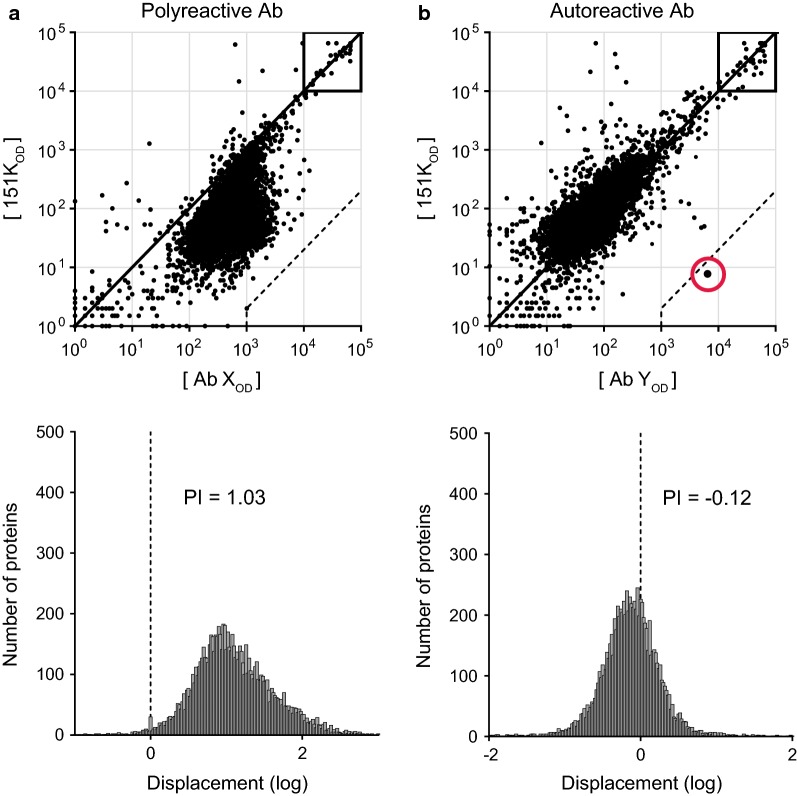
Protein microarray binding of hypothetical polyreactive (**a**) and autoreactive (**b**) bNAbs. *Top*, Protein arrays were blotted with a non-polyreactive control Ab (151K, *A* and *B*), Ab X (*A*) or Ab Y (*B*). Axis values represent the relative fluorescence signal intensity in the 151K array (y-axis) or the test Ab array (x-axis). Each dot represents an individual target protein. The diagonal line indicates equal binding by the two comparators. The dashed lines mark the cutoff for autoreactivity, set at 500-fold higher binding by the test Ab than by the control Ab. The red circle denotes an autoantigen bound ≥ 500-fold more avidly by Ab X than by the control Ab. *Bottom*, Histogram showing the displacement of each protein from the diagonal (*top*). The bin size is 0.02. Positive displacement indicates stronger binding by the test Ab than by 151K. The polyreactivity index (PI) is the Gaussian mean of all displacement values. The threshold of polyreactivity, set at PI = 0.21, is equivalent to twofold stronger overall binding by the test Ab than the control Ab

In the most general terms, *polyreactive* Abs are those that promiscuously bind apparently unrelated self- and/or foreign-antigens, while *autoreactive* Abs specifically bind one or few self-epitopes. Poly- and autoreactivity in Abs are empirically defined. One method defines *autoreactivity* as the ability of an Ab to bind any self-antigen, and defines *polyreactivity* as the ability to bind (in ELISA) two or more antigens from a set list that generally includes single-stranded DNA, double-stranded DNA (dsDNA), insulin, lipopolysaccharide, and keyhole limpet hemocyanin [16, 17]. Another method, established by our laboratory, determines poly- and autoreactivity by applying the Ab of interest together with a non-polyreactive control Ab to a microarray that displays > 9400 human proteins [18, 19]. Ab binding strength to each protein target is measured as fluorescence intensity, and if the averaged binding intensity over all arrayed proteins (i.e., mean fluorescence intensity; MFI) of the test Ab is > twofold greater than the MFI of the control Ab, then the experimental Ab is considered polyreactive (Fig. 1a) [19]. Non-polyreactive test Abs that bind a self-protein in the array with > 500-fold higher avidity than the control Ab are considered autoreactive (Fig. 1b) [19]. Notably, some polyreactive Abs also bind autoantigens with > 500-fold higher avidity than the control. However, for simplicity, we reserve the term *autoreactive* to describe non-polyreactive Abs, since substantial cumulative autoreactivity is already implied for Abs labeled *polyreactive* [19].

That many B-cell receptors (BCRs) recognize self-antigens is an inevitable by-product of the extraordinary diversity of BCRs generated during B-cell development. Indeed, ~ 75% of newly assembled human BCRs react with self-antigens [16]. This prevalent autoreactivity is potentially dangerous to the host, as evidenced by the generation of pathologic autoantibodies in many autoimmune diseases [20, 21]. Thus, mechanisms of immunological tolerance eliminate or silence autoreactive B cells at discrete checkpoints during B-cell development. At each checkpoint, autoreactive B cells are purged by clonal deletion (i.e., apoptosis) [22–24]; modified by receptor editing, in which continued V(D)J recombination alters BCR specificity [25, 26]; or rendered anergic (i.e., highly resistant to BCR stimulation and plasmacytic differentiation) [27, 28]. In consequence, the frequency of autoreactive B cells is reduced from 75% in the early immature B-cell stage to ~ 20% among mature B cells in healthy humans [16]. Concomitantly, there are dramatic reductions in the frequencies of polyreactive BCRs as well as those with a long third complementarity determining region in the Ab heavy chain (HCDR3) [16, 29, 30], suggesting that these properties are linked to autoreactivity and are efficiently counter-selected by immune tolerance controls. Finally, autoreactive BCRs also may be generated by V(D)J somatic hypermutation (SHM) in germinal center (GC) B cells [31]. These newly autoreactive GC B cells may be culled or silenced by apoptosis and other means [31–37], or else may be “redeemed” from negative selection by ongoing SHM that abolishes autospecificity [38–41]. Thus, healthy individuals constrain the B-cell repertoire to avoid generating potentially pathogenic self-reactive humoral responses.

The necessary evil of immunological tolerance is that it creates “holes” in the BCR repertoire. By having vulnerable epitopes mimic the molecular structures of host antigens, pathogens can exploit these holes and evade humoral responses [42]. In the following sections, we discuss (1) the evidence of molecular mimicry by HIV-1 broadly neutralizing epitopes, (2) the role of immune tolerance controls in suppressing the generation of bNAbs, and (3) possible strategies for accessing the forbidden BCR repertoire to achieve broad protection during HIV-1 vaccination.

## Immune tolerance blocks bNAb generation

HIV-1 bNAbs each exhibit at least one of these uncommon traits: poly- or autoreactivity, a long HCDR3, and/or extraordinary frequencies of V(D)J mutations. It is these features that suggest the hypothesis that immunological tolerance disfavors bNAb generation [13, 14]. Poly- or autoreactive B-cell antigen receptors (BCRs) are eliminated from the primary repertoire at central and peripheral tolerance checkpoints [16, 43, 44], and there is evidence that BCRs with long HCDR3s—which themselves are frequently poly- or autoreactive—are also filtered from the repertoire during B-cell development [29, 30]. While the impetus for extensive somatic hypermutation in bNAbs is unclear, it is plausible that in the absence of competition, B cells with initially poor binding to broadly neutralizing epitopes undergo tortuous and prolonged affinity maturation pathways until broad neutralization is achieved.

Initial evidence for the tolerance hypothesis arose from the discovery that some bNAbs, including 2F5 and 4E10, cross-react with self-lipids (e.g., cardiolipin) and various self-protein antigens in vitro [14]. The next test of the hypothesis was to determine whether bNAb autoreactivity is physiologically relevant for B-cell development, i.e., whether tolerance mechanisms counterselect developing B cells expressing mature bNAbs or their precursors. This was directly tested in knockin (KI) mice expressing the rearranged heavy- and light-chain (HC + LC) variable regions of mature bNAbs or their unmutated germline (gl) precursors. In support of the tolerance hypothesis, mice expressing the HC + LC of 2F5, gl2F5, 4E10, gl3BNC60, or HC of 2F5 or gl3BNC60 [5, 45–50] exhibited one or more traits—including marked clonal deletion of developing B cells, extensive BCR editing, and anergy in peripheral B cells—that define the tolerance controls observed in mice that express transgenic autoreactive BCRs [22, 23, 25, 26, 28, 51, 52]. Thus, the autoreactivity of bNAbs and their germline precursors is, in many cases, sufficient to proscribe normal development of B cells bearing these BCRs.

Knowing that immunological tolerance mechanisms often prohibit the development of B cells expressing mature or precursor bNAbs, our lab set out to isolate and identify the autoantigens recognized by bNAbs. In early studies, we used 2F5 and 4E10 to immunoprecipitate antigens from human cell lysates, and then identified the precipitated targets by peptide mass fingerprinting, followed by stringent immunoassays to filter the candidate list [18]. In this way, kynureninase (KYNU) was identified as the primary self-antigen bound by mature and gl2F5 bnAbs, whereas splicing factor 3b subunit 3 (SF3B3) was the principal target of 4E10 [18]. Strikingly, the complete 2F5 linear epitope (ELDKWA) is shared by HIV-1 Env and the known KYNU orthologs in most mammals, with the notable exception of opossums, which carry a point mutation in KYNU that abolishes 2F5 binding [18]. Accordingly, immunization of opposums resulted in ELDKWA-specific serum Ab titers that were ≥ 100-fold higher than in immunized mice; however, immunization did not generate opossum antibodies to the adjacent 4E10 epitope, consistent with the high degree of conservation between human and opossum SF3B3 [18]. That opposums can generate 2F5-like antibodies is not due to intrinsically longer HCDR3 segments in opossum antibodies, since the average HCDR3 length is equivalent (12–13 amino acids) in opossum and mouse BCRs [53, 54]. Rather, mice also have in their pre-tolerance BCR repertoire the latent capacity to respond to the ELDKWA epitope, and this specificity is eliminated by immunological tolerance. This was demonstrated in animals reconstituted with B cells that had bypassed central tolerance via an in vitro culture system [55]. After immunization with MPER peptide, reconstituted mice formed robust GC responses, whereas control animals did not [55]. Moreover, after secondary immunization, the MPER-specific serum IgG response was 12-fold higher in reconstituted mice than in controls [55]. Recently, we have determined that BCR specificity for KYNU and Env are almost perfectly correlated in 2F5 HC + LC KI mice: B cells that bind both KYNU and Env exist only prior to the first tolerance checkpoint or in a fraction of the peripheral IgM^−^IgD^+^ anergic B-cell pool, whereas no post-tolerance checkpoint mature B cell reacts with either KYNU or Env (Finney et al., manuscript in preparation). These data support the hypothesis that at least some broadly neutralizing viral epitopes avoid the immune response by mimicking host antigens and thereby hiding in “holes” created in the B-cell repertoire by immunological tolerance controls.

To determine whether poly/autoreactivity is linked with broadly neutralizing activity, rather than being merely a product of chronic infection (e.g., persistent inflammation, prolonged Ag exposure, etc.) [56–58], we used microarrays displaying > 9400 human proteins to screen panels of bNAbs and non-broadly neutralizing Abs (nNAbs, including non-neutralizing and autologous neutralizing Abs) alongside a non-polyreactive control Ab [18, 19]. We defined *polyreactive* Abs as those whose averaged array binding was > twofold greater than the control Ab, whereas *autoreactive* Abs were non-polyreactive Abs that bound at least one self-protein with > 500-fold higher avidity than the control Ab (Fig. 1) [19]. Using these criteria, we found that ~ 20% (2/9) of nNAbs were poly- or autoreactive [19], which is indistinguishable from the frequency of poly- and autoreactive B cells found among mature peripheral B cells in healthy humans [16]. In contrast, ~ 60% (13/22) of bNAbs were poly- or autoreactive, including ≥ 1 polyreactive bNAb in each of four major bNAb classes: CD4 binding-site, membrane-proximal external region (MPER), variable loops 1 and 2, and variable loop-associated glycan [19]. Importantly, bNAbs were also significantly enriched for poly/autoreactivity compared to the nNAbs isolated from infected patients (i.e., excluding nNAbs arising from vaccination) [19]. Thus, bNAb poly/autoreactivity is *not* a product of the infection milieu. Moreover, while the average frequency of V_H_ somatic mutations was substantially higher in bNAbs (20.5%) than in nNAbs (10%), SHM was not correlated with poly- or autoreactivity [19]. Likewise, whereas the average HCDR3 length in bNAbs (19.4 amino acids) was substantially longer than in nNAbs (14.7 amino acids), HCDR3 length did not correlate with poly- or autoreactivity. These data support that poly/autoreactivity is intrinsically linked to broadly neutralizing activity.

Notably, ~ 40% of bNAbs were neither poly- nor autoreactive when assessed for self-protein binding, raising the question of why they remain difficult to elicit. The protein array likely underestimates poly/autoreactivity, since some bNAbs engage non-protein self-molecules, e.g., PGT121 avidly binds self-glycans, even in the absence of protein determinants [19, 59–61]. Additionally, there are other proposed barriers to bNAb generation, including the sparsity of Env spikes on virions [62–64], conformational masking of broadly neutralizing epitopes [65, 66], immunological dominance of non-broadly neutralizing epitopes [1], and the requirement of some bNAb lineages for specific V-, D-, or J-gene allelic variants [67].

## Implications for vaccine design

In light of the role that immunological tolerance plays in barring the generation of many bNAbs, there are at least two potential strategies for a universal HIV-1 vaccine. One tactic is to work within the constraints of tolerance controls to elicit only those types of bNAbs not proscribed by immune tolerance. The second approach would be to design an immunization regimen that modulates or “breaks” tolerance to gain access to bNAb precursors in the forbidden repertoire. The former strategy, unlike the latter, carries no additional risk of developing autoimmune disease, and therefore is likely to face fewer barriers to regulatory approval and wide use. However, the potential shortcoming of this method is that it must achieve neutralization by targeting only a subset of vulnerable epitopes. In consequence, bNAbs would have to arise from an even smaller pool of already rare precursors. This limitation could further confound vaccination efforts, since precursor cell frequency may be an important determinant of B-cell competitiveness in anti-Env humoral responses [68, 69], and variability in the human BCR repertoire might preclude the generation of certain bNAb lineages in individuals lacking the required V-, D-, or J-gene allelic variants [12, 67]. However, these remain open questions and are potentially surmountable obstacles.

The second strategy—to break tolerance—has been recently attempted, with some success. 2F5 KI mice were repeatedly immunized with MPER peptide-conjugated liposomes (engineered to mimic the MPER epitopes present on virions) and TLR agonists [5, 70]. In 2F5 HC + LC KI mice, this method successfully overcame B-cell anergy to activate and expand populations of MPER-binding B cells, and also elicited substantial serum titers of MPER-specific neutralizing IgG [70]. Similarly, in gl2F5 KI mice, MPER-liposome vaccination induced selective proliferation of MPER-specific B cells; however, it failed to induce class-switch recombination and somatic hypermutation, and generated poor serum titers of anti-MPER IgM [5]. Likewise, in gl3BNC60 HC + LC KI mice, only highly multimerized immunogen (rather than trimeric immunogen) could reliably elicit serum antibody responses specific for the CD4 binding site [46]. However, activated glBNC60 HC + LC KI B cells harbored few—if any—V(D)J mutations [46]. Promisingly, immunization of macaques with MPER liposomes and TLR agonists generated serum Ab responses to the ELDKWA epitope in KYNU and MPER [5], indicating that appropriate vaccination regimens may break tolerance to self-antigens and permit Ab responses to conserved HIV-1 epitopes that mimic these antigens. Unfortunately, the MPER-binding antibodies had limited neutralization potency because the vaccine-induced SHM did not achieve the degree of HCDR3 hydrophobicity required for effective interaction with virion lipids and broad HIV-1 neutralization [5].

We propose that transient relaxation of tolerance controls might open an additional avenue to the establishment of broad, durable humoral protection to HIV-1. One way to accomplish this would be administration of hydroxychloroquine (Plaquenil™), an inexpensive and widely used antimalarial drug, to inhibit endosome acidification and reduce the stringency of the central tolerance checkpoint [71]. Our lab has shown that such treatment results in a reduced counterselection of autoreactive immature/transitional B cells in KI mice expressing the 2F5 HC + LC or the dsDNA-specific 3H9 BCR [71]. A second potential target for pharmacological modulation is the Cbl-b and c-Cbl ubiquitin ligases, which appear to enforce B-cell anergy [72]. Temporarily interfering with the Cbl proteins’ function might facilitate activation of autoreactive bNAb precursors. We emphasize here that these treatments would have to be transient and carefully timed in conjunction with vaccination, since extended relaxation of tolerance controls could result in autoimmune disease [72]. However, judicious use of hydroxychloroquine and as-yet-untested inhibitors of peripheral and GC tolerance controls might be a generally applicable tactic for increasing the frequency of peripheral bNAb precursors and enabling their maturation into potent bNAbs.

As we noted briefly above, an important consideration (and possible downfall) of any vaccine strategy to intentionally elicit autoreactive antibodies is the potential for increased risk of autoimmune disease. This concern is not without merit, as 4E10 has some anti-coagulant activity and modestly prolonged the activated partial thromboplastin time in HIV-1 patients receiving passive 4E10 immunotherapy [14, 73]. However, passive 4E10 treatment was otherwise well tolerated (as was infusion of 2F5, which did not affect coagulation), and the risk of thrombotic complications from 4E10 immunotherapy was deemed low [73]. Additional experiments in mice and macaques demonstrated that passively transferred 2F5 (or 2F5-like Abs raised by MPER-liposome vaccination) do not inhibit KYNU activity, alter tryptophan metabolism, nor produce other obvious side effects [74]. Therefore, while any vaccine regimen designed to generate autoreactive bNAbs would have to be evaluated with extra rigor to assure safety, host mimicry by many bNAb epitopes does not a priori disqualify this immunization strategy.

A final possibility for consideration is that of “clonal redemption” of autoreactive bNAb precursors through mutation away from self-reactivity during GC responses [40, 41]. Proof-of-concept studies by Goodnow and colleagues in mice suggest that this may be a relevant strategy for eliciting HIV-1 bNAbs to epitopes that imperfectly mimic host structures [39]. In mice expressing a mutant form of hen egg lysozyme (HEL^3X^) as a ubiquitous neo-autoantigen, HEL^3X^-specific B cells exhibited an anergic phenotype [39]. However, immunization with particulate immunogens expressing high densities of the closely related antigen, duck egg lysozyme (DEL), successfully recruited anergic HEL^3X^-specific B cells into GCs, where SHM and antigen-driven selection enriched clonal lineages with reduced affinity for self-antigen (HEL^3X^) and increased affinity for foreign antigen (DEL) [39]. Importantly, clones with enhanced binding to DEL (and diminished binding to HEL^3X^) could differentiate into memory B cells and Ab-secreting plasma cells. It will be exciting to determine whether this mechanism could also redeem autoreactive bNAb precursors. For example, assuming that the 2F5 nominal epitope (ELDKWA) present in Env and KYNU can be discriminated by minor structural differences, the right immunization regimen might induce gl2F5 to undergo affinity maturation to produce a mature bNAb that binds Env with high affinity and is no longer subject to stringent tolerance controls [5].

## Concluding remarks

B-cell tolerance controls are necessary to prevent the generation of self-antibodies and autoimmune disease. However, tolerance creates empty spaces in the Ab repertoire and these “holes” can be exploited by pathogens whose vulnerable epitopes structurally mimic self-antigens. From structural/biochemical studies of bNAbs and the generation of bNAb-knockin mice, it is now clear that HIV-1 is such a pathogen, disguising conserved, functionally important viral structures as various host proteins. In consequence, traditional vaccination strategies do not appear to be suitable for eliciting many bNAb lineages, since the B cells most fit to respond have been eliminated or silenced during their development, maturation, or antigen-driven expansion. These obstacles to effective HIV-1 vaccination, while serious, are not insurmountable. Recent evidence demonstrates that tolerance controls can be relaxed or broken to gain access to this forbidden Ab repertoire, without inducing autoimmune disease. Additionally, subtle structural differences between self-antigens and the foreign molecules that mimic them might permit initially autoreactive, physiologically silenced BCRs to be redeemed by V(D)J mutations that impair self-reactivity in GCs. Future studies in this field will likely focus on these aspects, particularly with regard to techniques for transiently modulating immunological tolerance in conjunction with vaccination, which have the potential to provide broad, durable protection.

## Abbreviations

Ab: antibody
BCR: B-cell receptor
bNAb: broadly neutralizing antibody
DEL: duck egg lysozyme
dsDNA: double-stranded DNA
Env: envelope protein
GC: germinal center
gl: germline
HC: heavy chain
HCDR3: heavy chain third complementarity-determining region
HEL^3x^: triply mutated hen egg lysozyme
KI: knockin
KYNU: kynureninase
LC: light chain
MFI: mean fluorescence intensity
MPER: membrane-proximal external region
nNAb: non-broadly neutralizing antibody
SF3B3: splicing factor 3b subunit 3
SHM: somatic hypermutation

## References

[1] Mascola JR, Haynes BF (2013). HIV-1 neutralizing antibodies: understanding nature’s pathways. Immunol Rev.

[2] Gray ES, Madiga MC, Hermanus T, Moore PL, Wibmer CK, Tumba NL, Werner L, Mlisana K, Sibeko S, Williamson C (2011). The neutralization breadth of HIV-1 develops incrementally over four years and is associated with CD4^+^ T cell decline and high viral load during acute infection. J Virol.

[3] Mikell I, Sather DN, Kalams SA, Altfeld M, Alter G, Stamatatos L (2011). Characteristics of the earliest cross-neutralizing antibody response to HIV-1. PLoS Pathog.

[4] Hraber P, Seaman MS, Bailer RT, Mascola JR, Montefiori DC, Korber BT (2014). Prevalence of broadly neutralizing antibody responses during chronic HIV-1 infection. AIDS.

[5] Zhang R, Verkoczy L, Wiehe K, Munir Alam S, Nicely NI, Santra S, Bradley T, Pemble CW, Zhang J, Gao F (2016). Initiation of immune tolerance-controlled HIV gp41 neutralizing B cell lineages. Sci Transl Med.

[6] Briney B, Sok D, Jardine JG, Kulp DW, Skog P, Menis S, Jacak R, Kalyuzhniy O, de Val N, Sesterhenn F (2016). Tailored immunogens direct affinity maturation toward HIV neutralizing antibodies. Cell.

[7] Escolano A, Steichen JM, Dosenovic P, Kulp DW, Golijanin J, Sok D, Freund NT, Gitlin AD, Oliveira T, Araki T (2016). Sequential immunization elicits broadly neutralizing anti-HIV-1 antibodies in Ig knockin mice. Cell.

[8] Xu K, Acharya P, Kong R, Cheng C, Chuang GY, Liu K, Louder MK, O’Dell S, Rawi R, Sastry M (2018). Epitope-based vaccine design yields fusion peptide-directed antibodies that neutralize diverse strains of HIV-1. Nat Med.

[9] Dubrovskaya V, Guenaga J, de Val N, Wilson R, Feng Y, Movsesyan A, Karlsson Hedestam GB, Ward AB, Wyatt RT (2017). Targeted N-glycan deletion at the receptor-binding site retains HIV Env NFL trimer integrity and accelerates the elicited antibody response. PLoS Pathog.

[10] Burton DR, Desrosiers RC, Doms RW, Koff WC, Kwong PD, Moore JP, Nabel GJ, Sodroski J, Wilson IA, Wyatt RT (2004). HIV vaccine design and the neutralizing antibody problem. Nat Immunol.

[11] Kwong PD, Mascola JR, Nabel GJ (2012). Rational design of vaccines to elicit broadly neutralizing antibodies to HIV-1. Cold Spring Harb Perspect Biol.

[12] Haynes BF, Kelsoe G, Harrison SC, Kepler TB (2012). B-cell-lineage immunogen design in vaccine development with HIV-1 as a case study. Nat Biotechnol.

[13] Haynes BF, Moody MA, Verkoczy L, Kelsoe G, Alam SM (2005). Antibody polyspecificity and neutralization of HIV-1: a hypothesis. Hum Antibodies.

[14] Haynes BF, Fleming J, St Clair EW, Katinger H, Stiegler G, Kunert R, Robinson J, Scearce RM, Plonk K, Staats HF (2005). Cardiolipin polyspecific autoreactivity in two broadly neutralizing HIV-1 antibodies. Science.

[15] Kelsoe G, Haynes BF (2017). Host controls of HIV broadly neutralizing antibody development. Immunol Rev.

[16] Wardemann H, Yurasov S, Schaefer A, Young JW, Meffre E, Nussenzweig MC (2003). Predominant autoantibody production by early human B cell precursors. Science.

[17] Mouquet H, Scheid JF, Zoller MJ, Krogsgaard M, Ott RG, Shukair S, Artyomov MN, Pietzsch J, Connors M, Pereyra F (2010). Polyreactivity increases the apparent affinity of anti-HIV antibodies by heteroligation. Nature.

[18] Yang G, Holl TM, Liu Y, Li Y, Lu X, Nicely NI, Kepler TB, Alam SM, Liao HX, Cain DW (2013). Identification of autoantigens recognized by the 2F5 and 4E10 broadly neutralizing HIV-1 antibodies. J Exp Med.

[19] Liu M, Yang G, Wiehe K, Nicely NI, Vandergrift NA, Rountree W, Bonsignori M, Alam SM, Gao J, Haynes BF, Kelsoe G (2015). Polyreactivity and autoreactivity among HIV-1 antibodies. J Virol.

[20] Rowley MJ, Whittingham SF (2015). The role of pathogenic autoantibodies in autoimmunity. Antibodies.

[21] Suurmond J, Diamond B (2015). Autoantibodies in systemic autoimmune diseases: specificity and pathogenicity. J Clin Investig.

[22] Nemazee DA, Burki K (1989). Clonal deletion of B lymphocytes in a transgenic mouse bearing anti-MHC class I antibody genes. Nature.

[23] Hartley SB, Crosbie J, Brink R, Kantor AB, Basten A, Goodnow CC (1991). Elimination from peripheral lymphoid tissues of self-reactive B lymphocytes recognizing membrane-bound antigens. Nature.

[24] Erikson J, Radic MZ, Camper SA, Hardy RR, Carmack C, Weigert M (1991). Expression of anti-DNA immunoglobulin transgenes in non-autoimmune mice. Nature.

[25] Gay D, Saunders T, Camper S, Weigert M (1993). Receptor editing: an approach by autoreactive B cells to escape tolerance. J Exp Med.

[26] Tiegs SL, Russell DM, Nemazee D (1993). Receptor editing in self-reactive bone marrow B cells. J Exp Med.

[27] Nossal GJ, Pike BL (1980). Clonal anergy: persistence in tolerant mice of antigen-binding B lymphocytes incapable of responding to antigen or mitogen. Proc Natl Acad Sci USA.

[28] Goodnow CC, Crosbie J, Jorgensen H, Brink RA, Basten A (1989). Induction of self-tolerance in mature peripheral B lymphocytes. Nature.

[29] Larimore K, McCormick MW, Robins HS, Greenberg PD (2012). Shaping of human germline IgH repertoires revealed by deep sequencing. J Immunol.

[30] Meffre E, Milili M, Blanco-Betancourt C, Antunes H, Nussenzweig MC, Schiff C (2001). Immunoglobulin heavy chain expression shapes the B cell receptor repertoire in human B cell development. J Clin Investig.

[31] DeFranco AL (2016). Germinal centers and autoimmune disease in humans and mice. Immunol Cell Biol.

[32] Chan TD, Wood K, Hermes JR, Butt D, Jolly CJ, Basten A, Brink R (2012). Elimination of germinal-center-derived self-reactive B cells is governed by the location and concentration of self-antigen. Immunity.

[33] Han S, Zheng B, Dal Porto J, Kelsoe G (1995). In situ studies of the primary immune response to (4-hydroxy-3-nitrophenyl)acetyl. IV. Affinity-dependent, antigen-driven B cell apoptosis in germinal centers as a mechanism for maintaining self-tolerance. J Exp Med.

[34] Shokat KM, Goodnow CC (1995). Antigen-induced B-cell death and elimination during germinal-centre immune responses. Nature.

[35] Pulendran B, Kannourakis G, Nouri S, Smith KG, Nossal GJ (1995). Soluble antigen can cause enhanced apoptosis of germinal-centre B cells. Nature.

[36] Pulendran B, Smith KG, Nossal GJ (1995). Soluble antigen can impede affinity maturation and the germinal center reaction but enhance extrafollicular immunoglobulin production. J Immunol.

[37] Wong EB, Soni C, Chan AY, Domeier PP, Abraham T, Limaye N, Khan TN, Elias MJ, Chodisetti SB (2015). B cell-intrinsic CD84 and Ly108 maintain germinal center B cell tolerance. J Immunol.

[38] Mayer CT, Gazumyan A, Kara EE, Gitlin AD, Golijanin J, Viant C, Pai J, Oliveira TY, Wang Q, Escolano A (2017). The microanatomic segregation of selection by apoptosis in the germinal center. Science.

[39] Burnett DL, Langley DB, Schofield P, Hermes JR, Chan TD, Jackson J, Bourne K, Reed JH, Patterson K, Porebski BT (2018). Germinal center antibody mutation trajectories are determined by rapid self/foreign discrimination. Science.

[40] Reed JH, Jackson J, Christ D, Goodnow CC (2016). Clonal redemption of autoantibodies by somatic hypermutation away from self-reactivity during human immunization. J Exp Med.

[41] Sabouri Z, Schofield P, Horikawa K, Spierings E, Kipling D, Randall KL, Langley D, Roome B, Vazquez-Lombardi R, Rouet R (2014). Redemption of autoantibodies on anergic B cells by variable-region glycosylation and mutation away from self-reactivity. Proc Natl Acad Sci USA.

[42] Bowes T, Wagner ER, Boffey J, Nicholl D, Cochrane L, Benboubetra M, Conner J, Furukawa K, Furukawa K, Willison HJ (2002). Tolerance to self gangliosides is the major factor restricting the antibody response to lipopolysaccharide core oligosaccharides in *Campylobacter jejuni* strains associated with Guillain-Barré syndrome. Infect Immun.

[43] Pelanda R, Torres RM (2012). Central B-cell tolerance: where selection begins. Cold Spring Harb Perspect Biol.

[44] Nemazee D (2017). Mechanisms of central tolerance for B cells. Nat Rev Immunol.

[45] Dosenovic P, von Boehmer L, Escolano A, Jardine J, Freund NT, Gitlin AD, McGuire AT, Kulp DW, Oliveira T, Scharf L (2015). Immunization for HIV-1 broadly neutralizing antibodies in human Ig knockin mice. Cell.

[46] McGuire AT, Gray MD, Dosenovic P, Gitlin AD, Freund NT, Petersen J, Correnti C, Johnsen W, Kegel R, Stuart AB (2016). Specifically modified Env immunogens activate B-cell precursors of broadly neutralizing HIV-1 antibodies in transgenic mice. Nat Commun.

[47] Verkoczy L, Diaz M, Holl TM, Ouyang YB, Bouton-Verville H, Alam SM, Liao HX, Kelsoe G, Haynes BF (2010). Autoreactivity in an HIV-1 broadly reactive neutralizing antibody variable region heavy chain induces immunologic tolerance. Proc Natl Acad Sci USA.

[48] Verkoczy L, Chen Y, Bouton-Verville H, Zhang J, Diaz M, Hutchinson J, Ouyang YB, Alam SM, Holl TM, Hwang KK (2011). Rescue of HIV-1 broad neutralizing antibody-expressing B cells in 2F5 VHx VL knockin mice reveals multiple tolerance controls. J Immunol.

[49] Chen Y, Zhang J, Hwang KK, Bouton-Verville H, Xia SM, Newman A, Ouyang YB, Haynes BF, Verkoczy L (2013). Common tolerance mechanisms, but distinct cross-reactivities associated with gp41 and lipids, limit production of HIV-1 broad neutralizing antibodies 2F5 and 4E10. J Immunol.

[50] Doyle-Cooper C, Hudson KE, Cooper AB, Ota T, Skog P, Dawson PE, Zwick MB, Schief WR, Burton DR, Nemazee D (2013). Immune tolerance negatively regulates B cells in knock-in mice expressing broadly neutralizing HIV antibody 4E10. J Immunol.

[51] Nemazee D, Buerki K (1989). Clonal deletion of autoreactive B lymphocytes in bone marrow chimeras. Proc Natl Acad Sci USA.

[52] Goodnow CC, Crosbie J, Adelstein S, Lavoie TB, Smith-Gill SJ, Brink RA, Pritchard-Briscoe H, Wotherspoon JS, Loblay RH, Raphael K (1988). Altered immunoglobulin expression and functional silencing of self-reactive B lymphocytes in transgenic mice. Nature.

[53] Wang X, Sharp AR, Miller RD (2012). Early postnatal B cell ontogeny and antibody repertoire maturation in the opossum, *Monodelphis domestica*. PLoS ONE.

[54] Kuraoka M, Schmidt AG, Nojima T, Feng F, Watanabe A, Kitamura D, Harrison SC, Kepler TB, Kelsoe G (2016). Complex antigens drive permissive clonal selection in germinal centers. Immunity.

[55] Holl TM, Yang G, Kuraoka M, Verkoczy L, Alam SM, Moody MA, Haynes BF, Kelsoe G (2014). Enhanced antibody responses to an HIV-1 membrane-proximal external region antigen in mice reconstituted with cultured lymphocytes. J Immunol.

[56] Bonsignori M, Wiehe K, Grimm SK, Lynch R, Yang G, Kozink DM, Perrin F, Cooper AJ, Hwang KK, Chen X (2014). An autoreactive antibody from an SLE/HIV-1 individual broadly neutralizes HIV-1. J Clin Investig.

[57] Ditzel HJ, Itoh K, Burton DR (1996). Determinants of polyreactivity in a large panel of recombinant human antibodies from HIV-1 infection. J Immunol.

[58] Satoh M, Kuroda Y, Yoshida H, Behney KM, Mizutani A, Akaogi J, Nacionales DC, Lorenson TD, Rosenbauer RJ, Reeves WH (2003). Induction of lupus autoantibodies by adjuvants. J Autoimmun.

[59] Mouquet H, Scharf L, Euler Z, Liu Y, Eden C, Scheid JF, Halper-Stromberg A, Gnanapragasam PN, Spencer DI, Seaman MS (2012). Complex-type N-glycan recognition by potent broadly neutralizing HIV antibodies. Proc Natl Acad Sci USA.

[60] Walker LM, Huber M, Doores KJ, Falkowska E, Pejchal R, Julien JP, Wang SK, Ramos A, Chan-Hui PY, Moyle M (2011). Broad neutralization coverage of HIV by multiple highly potent antibodies. Nature.

[61] Julien JP, Sok D, Khayat R, Lee JH, Doores KJ, Walker LM, Ramos A, Diwanji DC, Pejchal R, Cupo A (2013). Broadly neutralizing antibody PGT121 allosterically modulates CD4 binding via recognition of the HIV-1 gp120 V3 base and multiple surrounding glycans. PLoS Pathog.

[62] Zanetti G, Briggs JA, Grunewald K, Sattentau QJ, Fuller SD (2006). Cryo-electron tomographic structure of an immunodeficiency virus envelope complex in situ. PLoS Pathog.

[63] Zhu P, Liu J, Bess J, Chertova E, Lifson JD, Grise H, Ofek GA, Taylor KA, Roux KH (2006). Distribution and three-dimensional structure of AIDS virus envelope spikes. Nature.

[64] Burton DR, Hangartner L (2016). Broadly neutralizing antibodies to HIV and their role in vaccine design. Annu Rev Immunol.

[65] Kwong PD, Doyle ML, Casper DJ, Cicala C, Leavitt SA, Majeed S, Steenbeke TD, Venturi M, Chaiken I, Fung M (2002). HIV-1 evades antibody-mediated neutralization through conformational masking of receptor-binding sites. Nature.

[66] Munro JB, Gorman J, Ma X, Zhou Z, Arthos J, Burton DR, Koff WC, Courter JR, Smith AB, Kwong PD (2014). Conformational dynamics of single HIV-1 envelope trimers on the surface of native virions. Science.

[67] Alam SM, Liao HX, Dennison SM, Jaeger F, Parks R, Anasti K, Foulger A, Donathan M, Lucas J, Verkoczy L (2011). Differential reactivity of germline allelic variants of a broadly neutralizing HIV-1 antibody to a gp41 fusion intermediate conformation. J Virol.

[68] Abbott RK, Lee JH, Menis S, Skog P, Rossi M, Ota T, Kulp DW, Bhullar D, Kalyuzhniy O, Havenar-Daughton C (2018). Precursor frequency and affinity determine B cell competitive fitness in germinal centers, tested with germline-targeting HIV vaccine immunogens. Immunity.

[69] Dosenovic P, Kara EE, Pettersson AK, McGuire AT, Gray M, Hartweger H, Thientosapol ES, Stamatatos L, Nussenzweig MC (2018). Anti-HIV-1 B cell responses are dependent on B cell precursor frequency and antigen-binding affinity. Proc Natl Acad Sci USA.

[70] Verkoczy L, Chen Y, Zhang J, Bouton-Verville H, Newman A, Lockwood B, Scearce RM, Montefiori DC, Dennison SM, Xia SM (2013). Induction of HIV-1 broad neutralizing antibodies in 2F5 knock-in mice: selection against membrane proximal external region-associated autoreactivity limits T-dependent responses. J Immunol.

[71] Kuraoka M, Snowden PB, Nojima T, Verkoczy L, Haynes BF, Kitamura D, Kelsoe G (2017). BCR and endosomal TLR signals synergize to increase AID expression and establish central B cell tolerance. Cell Rep.

[72] Kitaura Y, Jang IK, Wang Y, Han YC, Inazu T, Cadera EJ, Schlissel M, Hardy RR, Gu H (2007). Control of the B cell-intrinsic tolerance programs by ubiquitin ligases Cbl and Cbl-b. Immunity.

[73] Vcelar B, Stiegler G, Wolf HM, Muntean W, Leschnik B, Mehandru S, Markowitz M, Armbruster C, Kunert R, Eibl MM, Katinger H (2007). Reassessment of autoreactivity of the broadly neutralizing HIV antibodies 4E10 and 2F5 and retrospective analysis of clinical safety data. Aids.

[74] Bradley T, Yang G, Ilkayeva O, Holl TM, Zhang R, Zhang J, Santra S, Fox CB, Reed SG, Parks R (2016). HIV-1 envelope mimicry of host enzyme kynureninase does not disrupt tryptophan metabolism. J Immunol.

